# Gender dynamics in local organizations: enhancing community participation for sustainable rural development in Indonesia

**DOI:** 10.3389/fsoc.2025.1673863

**Published:** 2026-01-16

**Authors:** Riri Amandaria, Rahim Darma, Sopian Tamrin, Rahmadanih Rahmadanih, Untari Untari

**Affiliations:** 1 Department of Anthropology and Sociology, University of Negeri Makassar, Makassar, Indonesia; 2 Department of Agricultural Socio-Economics, Laboratory of Agribusiness, University of Hasanuddin, Makassar, Indonesia; 3 Department of Agricultural Sciences, Faculty of Agriculture, University of Musamus, Merauke, Indonesia

**Keywords:** community participation, gender roles, institutional barriers, rural governance, women's leadership

## Abstract

Gender continues to influence participation in rural governance and economic development. This study examines how gender roles influence participation and leadership in local development organizations (LDOs) and local economic organizations (LEOs) in Ampekale Village, South Sulawesi, Indonesia. Using a qualitative case study design that combined in-depth interviews, focus group discussions, and participant observation, this research draws on insights from 24 key informants (12 men and 10 women), including village officials, organizational leaders, women's groups, NGO facilitators, and community members. The data were thematically coded and analyzed to capture the dynamics of gendered participation. The results show that men dominate leadership and infrastructure planning within LDOs, whereas women's involvement is often symbolic and restricted to welfare and social concerns. In LEOs, women are increasingly active in small-scale enterprises such as crab and snack production. Although these roles are aligned with domestic responsibilities, they enhance household bargaining power and expand women's visibility in governance. Men continue to lead physically demanding production and procurement, but women contribute substantially to value addition, marketing, and household welfare. The findings indicate that entrenched cultural norms, unequal access to resources, and limited leadership opportunities constrain women's influence. To foster equitable rural development, gender-sensitive reforms, such as quotas, participatory budgeting, training, and leadership mentoring are essential.

## 1 Introduction

Rural areas occupy a pivotal role in Indonesia's national development agenda, as they are central to achieving equity, integration, and inclusive growth, despite representing the lowest tier of governance. Through Law No. 32/2004 and Law No. 72/2005, villages have been granted the authority to manage their own development, creating opportunities for citizens to participate directly in governance and decision-making (Kamaludin, 2023; Shahid et al., 2022; Sobhaninia, 2023; Tselios and Rodríguez-Pose, 2022). Within this framework, promoting gender inclusion in local organizations and self-help groups emerges as a vital strategy for strengthening community-led empowerment and ensuring equitable and sustainable development outcomes (Hoesein et al., 2022; Klymchuk et al., 2022; Sulemana and Amakye, 2019). At the policy level, gender integration has been institutionalized through key regulations, such as Presidential Instruction (Inpres) No. 9 of 2000 on Gender Mainstreaming, which provides the foundation for incorporating gender perspectives into national and local development planning. This mandate has been reinforced by the establishment of the Ministry of Women's Empowerment and Child Protection *(Kementerian PPPA)* under Presidential Regulation No. 186 of 2024 and the Circular of the Minister of Women's Empowerment and Child Protection No. 1 of 2024, which calls for accelerated implementation of gender mainstreaming in all development policies to achieve gender equality.

The primary goal of Indonesian national development is to strengthen rural areas, which, although the lowest tier of governance, are central to achieving equity and integration in Indonesia. Law No. 32/2004 and Law No. 72/2005 grant villages the authority to manage their own development, enabling citizen participation in governance and decision making (Kamaludin, 2023; Shahid et al., 2022; Sobhaninia, 2023; Tselios and Rodríguez-Pose, 2022). Promoting gender inclusion in local organizations and self-help groups is a vital pathway to community-led empowerment and equitable growth (Hoesein et al., 2022; Klymchuk et al., 2022; Sulemana and Amakye, 2019).

The local organizations examined in this study operate as civil society organizations and community-based groups, including self-help collectives, farmer and fisher associations, women's groups, and village-representative bodies. These entities partner with village administrations rather than constituting formal government institutions in the region. They serve as intermediary actors that represent community interests, facilitate participatory processes, and complement the official functions of local government (Mahajan, 2021; Uphoff, 1993). Though lacking direct political authority, they are essential to grassroots governance, enabling collective action, creating platforms for negotiation, and strengthening social accountability (Silva et al., 2018; van Kempen, 2014). Their collaborative role with local government makes them especially significant for promoting inclusivity and gender equity, as civil society organizations are widely recognized as vital actors in building community empowerment and advancing participatory rural development (Cornwall and Rivas, 2015; Kabeer, 1999).

Ampekale Village in the Bontoa District of Maros District, South Sulawesi, offers a representatinct context for exploring these issues. With a population of 3,041 in 2023, the village reflects a dual socio-economic base shaped by its two subvillages. Binanga Sangkara, located along the shoreline, is dominated by capture fisheries and related activities, while Lalang Tedong functions as the administrative hub, providing governance and social services. Men are largely engaged in fishing, infrastructure, and raw material procurement, whereas women contribute significantly through processing, marketing, and small-scale enterprises, such as crab processing (BPS, 2024). Local organizations, including fishing and processing groups, support household livelihoods, whileAmpekale's proximity to the Maros District capital ensures accessibility to markets and services, making it a relevant site for examining gender and rural development participation.

Despite these opportunities, women's participation in governance and rural development remains limited. Limited education and technical knowledge hinder effective engagement (Ismail, 2022; Patil et al., 2023), while structural inequalities, resource restrictions, and entrenched discrimination weaken women's leadership potential (Agrawal et al., 2023; Kerr et al., 2013). Traditional gender norms further confine women to domestic roles, increasing their vulnerability to exclusion and gender-based violence, while limiting their representation in leadership positions (Lomazzi, 2023; Nnyombi et al., 2022; Rapinyana and Baratedi, 2023; Seema et al., 2023). To address these challenges, this study focuses on three interconnected aspects: the division of gender roles in local organizations, barriers to women's leadership, and policy impacts shaping participation in rural development. Examining these dimensions provides insights into the cultural and institutional dynamics that enable or hinder gender inclusion, offering evidence of more equitable and sustainable strategies.

This study draws on gender and participation theories that link power, agency, and inclusivity in governance. Gender roles are socially constructed and institutionalized, shaping access to resources and leadership opportunities (Cornwall, 2008; Kabeer, 1999). Participation is conceived not only as numerical inclusion but also as a meaningful influence in community processes (Agarwal, 2001, 2018). Therefore, local organizations are seen as critical spaces for negotiation, empowerment, and institutional transformation that advance women's leadership and strengthen community participation.

Rural development studies highlight the vital role of local organizations in enabling collective action, decision-making, and implementation of community initiatives (Elphina Nomabandla et al., 2023; Korten, 2018; Venus et al., 2017; Wanner and Wadham, 2015). Gender dynamics within these organizations are crucial, as inclusive participation leads to fairer resource distribution and stronger outcomes (Opare, 2007; Tanwir and Safdar, 2013). However, persistent barriers such as entrenched norms, limited resources, and traditional expectations continue to restrict women's involvement, underscoring the need for interventions that foster gender inclusivity (Awuni et al., 2022; Boasinke, 2021; Rakotoarison, 2024; Simatupang, 2022; Tsehay, 2020; Yount et al., 2024). Although the literature acknowledges the significance of gender and local organizations, there is little clarity on the specific mechanisms through which inclusivity can be achieved, and few studies have examined how gender intersects with other socio-economic factors in shaping participation.

This study investigated the roles and contributions of men and women in local organizations involved in rural development. It seeks to identify barriers to equitable participation, including entrenched norms and power imbalances, while assessing how gender-sensitive policies and inclusive practices can enhance women's engagement. It also aims to highlight the psychological and social benefits of inclusion, such as strengthened cohesion, trust, and collective commitment within communities. The findings provide insights into the dynamics of gendered participation, informing more targeted strategies for equity and collaboration. By clarifying the motivations, barriers, and contributions of men and women.

The study contributes to the broader discourse on gender and development, emphasizing inclusive approaches that value diverse perspectives and strengthen community resilience. Ultimately, this study seeks to advance sustainable and equitable rural development by fostering a culture of gender inclusivity and cooperation.

## 2 Research method

This study purposively selected Ampekale Village, Maros District, South Sulawesi, Indonesia, as the field site because of its dynamic development shaped by active local organizations. The village offers a relevant context for examining gender dynamics, given its dual socio-economic characteristics. In Binanga Sangkara, a fishery-based sub-village, women are central to processing, marketing, and small-scale enterprises, such as crab processing. In contrast, in Lalang Tedong, the administrative center, women participate more in governance and social services, although men still dominate decision-making. These contrasts highlight how location-specific socioeconomic and institutional features shape gendered opportunities, justifying Ampekale as a case study to explore the intersection of gender, local organizations, and rural development.

This study used a case study methodology that combined participant observation, in-depth interviews, and focus group discussions (FGDs). These qualitative techniques provide comprehensive insights into local organizational dynamics and gender participation. Structured questions guided formal and informal interactions with key informants from the organizational committees, target groups, and wider community. This approach generated contextual perspectives on gender roles and the effectiveness of development initiatives, capturing the complexities of community dynamics and gender relations in the process.

The analysis also considers the spatial variations in gender dynamics. In Binanga Sangkara, where fisheries dominate livelihoods, women play key roles in processing, marketing, and small-scale enterprises related to fish. Meanwhile, in Lalang Tedong, the administrative hub, women are more visible in governance and social services, although men continue to lead in strategic decisions. Recognizing these differences situates gender analysis within each sub-village's socioeconomic and institutional context.

The primary key informants chosen for this study included the village chief, local organization members, and community members who had knowledge of local organizations and rural development initiatives. The data collection process involved holding focus group discussions (FGDs) with the community to validate the information obtained from in-depth interviews. To ensure participant identity protection, the interviews meticulously documented, transcribed, and coded all the data collected from the FGDs and interviews. Furthermore, the study considers the varied gender composition of local organizations, recognizing its importance in understanding the complexities of community engagement and rural development efforts.

Table 1 shows that 24 key informants were purposively selected to capture diverse perspectives on gender participation and the role of local organizations in Ampekale Village. The informants included village government officials, leaders of local economic organizations, women's group members, NGO facilitators, and informal community leaders, representing both subvillages of Binanga Sangkara and Lalang Tedong. This composition ensured that voices from different socioeconomic and institutional contexts were reflected in the data. The number of informants was determined by the principle of data saturation, whereby interviews continued until recurring themes were identified and no substantial new insights were found (Guest et al., 2006; Hennink et al., 2017; Mason, 2010; Vasileiou et al., 2018). The purposive approach allowed for a balanced understanding of how gender roles, organizational structures, and community dynamics intersect to shape rural development outcomes.

**Table 1:** List and number of key informants categorized by status and gender.

**Informant**	**Status**	**Men**	**Women**	**Location**
Government body	Village head	1		Lalang Tedong
	Village secretary	1		Lalang Tedong
BPD	BPD chair	1		Lalang Tedong
	BPD vice chair	1		Lalang Tedong
	Member		2	Binanga Sangkara
NGO	Development planning facilitator	1		Lalang Tedong
	Business development facilitator	1		Lalang Tedong
Informal leader	Women leader		1	Lalang Tedong
	Local leader	1		Lalang Tedong
Economic Organization	Chair		1	Binanga Sangkara
	Secretary		1	Binanga Sangkara
	Members	1	1	Binanga Sangkara
Target group	Community members	4	4	Lalang tedong and Binanga Sangkara
Total		12	10	

The data encompassed community contributions to development activities, particularly infrastructure projects, as well as the roles played by local groups in rural development planning. It assesses the participation of both men and women in these groups and within broader planning processes. Secondary data obtained from village offices include historical records, demographic profiles, and lists of poor households. The analysis focused on Local Organization Development (LOD) and Non-Government Organizations (NGOs), with the Village Representative Body (VRB) VCI (Village Community Institute*-Lembaga Masyarakat Desa-LMD*) and Village Commnunity Security Institute (VCSI, *Lembaga Ketahan Masyarakat Desa-LKMD*) serving as part of the LOD and Yayasan Konservasi Laut (YKL) and Oxfam representing NGOs. Notably, YKL and Oxfam collaborated with CIDA (Canadian International Development Agency), thereby linking local initiatives with international support. This combination of primary and secondary sources provided a comprehensive perspective on rural development dynamics and the diverse contributions of stakeholders in the study area.

Qualitative analysis in this study focused on describing local organizations, their governance structures, and their functions, while examining the differentiated roles of men and women to understand how gender influences participation and decision-making. The analysis emphasized comparing gendered involvement to identify underlying patterns, barriers, and opportunities for inclusivity. By applying this approach, the study aimed to capture not only the formal aspects of institutional arrangements but also the social and cultural dynamics that shape organizational behavior and community participation.

## 3 Results

### 3.1 Local community organization

Local community organizations in the study area are comprised of Local Development Organizations (LDOs) and Local Economic Organizations (LEOs), whose membership consists of local residents and whose activities position them as important partners for both local governments and NGOs. The findings highlight how gender dynamics are shaped by the distinct socioeconomic and administrative context of the village. In Binanga Sangkara, a fishery-based sub-village, women assume central roles in fish processing, marketing, and small-scale enterprises, while in Lalang Tedong, the administrative center, women's participation is more evident in governance and social services, although leadership positions remain largely male dominated. These contrasts illustrate how local contexts create both opportunities and constraints for men and women's engagement in rural development, providing an important foundation for the more detailed analysis presented in the subsequent subsections.

#### 3.1.1 Local development organization (LDOs)

The VRB in Ampekale plays a central role in connecting the community with governance, drafting regulations, and overseeing development plans. Reinstated under Government Regulation No. 72/2005 and Village Law No. 6/2014, the VRB marked a shift toward participatory governance after earlier institutions such as the VCI (Village Community Institute*-Lembaga Masyarakat Desa-LMD*) and Village Commnunity Security Institute (VCSI, *Lembaga Ketahan Masyarakat Desa-LKMD*) failed to represent the local aspirations. Despite this progress, a gender imbalance persists only two of the nine members are women, with leadership roles held exclusively by men. Women's presence in village development planning forums (*Musrenbangdes-Musyawara Perencanaan Pembanguan Desa*) is often symbolic rather than substantive, particularly in decisions related to infrastructure and budgeting issues.

In practice, women's voices are typically confined to welfare and social issues, whereas decisions on budgets and infrastructure priorities continue to be shaped almost exclusively by male leaders. This imbalance illustrates the gap between formal representation and women being physically included in governance spaces—and actual influence, where their perspectives have a limited impact on how resources are allocated and development priorities are set. *Musrenbangdes* is the initial planning stage, carried out annually in January of the following year. The results of *Musrenbangdes* were brought to the sub-district level, then to the district/city level, and finally to the national level at the end of the year. All *Musrenbang* results were implemented the following year at each planning level.

The finding indicates that the VRB holds significant potential as a platform for inclusive development, but its effectiveness is constrained by persistent gender imbalances. Strengthening gender-inclusive policies within such institutions is therefore essential, and international evidence suggests that reforms such as quotas, gender-sensitive facilitation, and participatory budgeting can improve women's ability to influence decision-making processes. By situating the study's finding within this broader scholarship, the results affirm that while VRB is structurally positioned as a promising space for community participation, deliberate interventions are required to ensure women's contributions translate into meaningful influence rather than symbolic presence.

These results echo the wider critiques of decentralization and gendered governance, where women's roles in rural institutions are limited by structural and cultural barriers. Patriarchal norms, the double burden of domestic and productive work, and limited access to political education and leadership training continue to restrict women's strategic participation in politics. The Ampekale case shows how these barriers reproduce gender inequality in governance and constrain the transformative potential of participatory institutions.

This underrepresentation reflects structural and cultural barriers, including patriarchal norms that privilege male leadership, women's double burden of domestic and economic responsibilities, and limited access to political education and leadership training for women. These factors restrict women's opportunities to assume strategic roles and perpetuate inequalities in local governance. Therefore, strengthening gender-inclusive policies within the BPD is crucial to ensure that women's voices influence decision-making and promote equitable and sustainable rural development.

#### 3.1.2 Local economic organization

Local economic organizations (LEOs) in Ampekale Village, including farmers, fishing, and business groups, play a vital role in promoting rural development. Although development agents initiated these organizations, the local community has been instrumental in shaping and sustaining them. One notable example is the Ujung Parappa (UP) group established in 2011 through a partnership between *YKL*, Oxfam, and the CIDA. These organizations aim to improve the livelihoods of underprivileged fishing families, create job opportunities, and support community-led transformations.

A significant contribution comes from the women's group, consisting of fishermen's spouses who formed their own LEO, focused on crab processing. Since 2012, this group has played an active role in the local economy by using traditional low capital methods to generate income. Their participation highlights how women contribute to economic resilience by aligning their roles with social norms. The crab-processing industry has become a key part of the village economy, showing that even small-scale home-based activities can drive meaningful development when supported by a strong community organization.

The results show a clear gender-based division of labor in Ampekale's rural development initiatives, particularly in village infrastructure projects and local economic organizations (LEOs) in Indonesia. Men primarily engage in physically demanding and technically skilled tasks, such as digging, constructing irrigation systems, building farm roads, and providing tools and building supplies. One male informant explained this as follows:

*We usually handle heavy work, such as digging or carrying stones and cement. It is considered our responsibility as men*. (Male participant, Binanga Sangkara)

In contrast, women contribute to supportive and logistical roles, including preparing meals for workers and transporting lighter materials such as sand and water. As one woman emphasized,

*When the men are working on construction, we prepare food and sometimes help to carry water or small items. It is our way to support the work*. (Female participant, Lalang Tedong)

Although less visible, these contributions are essential for maintaining the smooth progress of development activities. Several participants underscored that such arrangements followed long-standing community norms regarding men's physical strength and women's caregiving responsibilities. A female informant noted:

*People here believe men are stronger, so they do the hard jobs, she said. Women help in ways that are closer to our daily work at home, like cooking or looking after needs*. (Female participant, Binanga Sangkara)

This evidence demonstrates how traditional perceptions of capability and appropriateness continue to shape the distribution of tasks. This finding suggests that participation is not only divided by gender but also justified through cultural expectations embedded in daily practices.

The division of labor within these rural organizations remains gendered but complementary. Men are mostly involved in fishing, raw material procurement, and production, whereas women are focused on processing, packaging, and marketing. Although men dominate leadership and physically demanding roles, women's contribution to value-adding and home-based enterprises is vital. The active role of the Ujung Parappa Group in village development planning further illustrates how grassroots economic initiatives can strengthen the local governance. Promoting gender-equitable participation and leadership within LEOs would enhance their effectiveness and support inclusive rural development.

### 3.2 Rural development activities: a gender-based analysis

The study shows that rural development in Ampekale Village involves clear gender-based roles, particularly in infrastructure projects facilitated by the Village Representative Board (BPD). As the key municipal body, the BPD manages community participation through *Musrenbangdes* forums that guide midterm development planning (RPJMDes). While men continue to dominate the board, with only two of the nine members being women, men and women make distinct contributions to the board. Men are generally responsible for heavy tasks, such as excavation, construction, and provision of materials. In contrast, women focus on logistical support, which includes preparing meals, providing water and transporting lighter materials.

This division also extends to development planning. Women typically raise household-level concerns, such as welfare, health, and livelihoods, whereas men tend to concentrate on infrastructure, including irrigation, roads, and public facilities. Although *Musrenbangdes* requires 30% female participation, women's voices remain stronger in domestic and social issues than in infrastructure priorities, reflecting the enduring gender norms in public decision-making.

Simultaneously, women's growing involvement in local economic organizations (LEOs) has begun to shift household and community power relations. Activities such as crab processing, snack production, and trading provide women with independent income, strengthening their bargaining power in household decisions regarding education and daily expenses. At the community level, participation in LEOs improves women's visibility and credibility, enabling them to negotiate more confidently with male leaders than before. While their authority over larger investments remains limited, these changes show increasing agency, as financial contributions enhance women's influence in both household and community governance.

Despite the division of labor, men's and women's complementary contributions are vital to rural development. Infrastructure projects rely on men's labor and technical skills, while women sustain efforts by managing daily logistics and community morale. For example, in mosque or road construction, men provide labor and materials, while women prepare meals and manage water and sand. Such collaboration illustrates how development is a collective process shaped by gender roles. Expanding women's participation in leadership and infrastructure planning could further enhance the inclusiveness and effectiveness of local development.

Table 2 highlights the gender-based division of labor in Ampekale's rural development efforts. Men take on physically demanding and skilled tasks—digging, building infrastructure, and supplying materials—that are essential for large-scale projects such as irrigation, farm roads, and mosque construction. Women provide crucial logistical support by preparing meals and transporting lighter materials, such as sand and water. Although less visible, these roles sustain the continuity of development work. This division reflects traditional norms that assign men to public physical labor and women to supportive caregiving functions.

**Table 2:** List of gender-based role development activities.

**Development activities**	**Input of men**	**Input of women**
Farm road	Supply of manual labor and tools for construction work such as excavation of stones and digging sand	Provision of food for workers
Irrigation	Supply of manual labor and tools for construction work such digging of stones and sand	Provision of food for workers
Foot path	Supply of manual labor and tools for construction work	Transportation of water and sand Provision of food for workers
Mosque construction	Supply of manual and skilled labor and tools for construction work and local materials such as stones, sands, wood, bamboo and water	Transportation of water and sand Provision of food for workers Cleaning of project site
Fresh water ponds	Supply of manual labor such as excavation of stones and removal of sand, provision of tools for construction	Transportation of water and sand Provision of food for workers
Sub-district public health service	Supply of manual labor and tools for construction work	Transportation of water and sand Provision of food for workers Cleaning of project site
Gathering for house construction	Supply of manual and skilled labor and tools for construction work	Provision of food for workers Cleaning of project site

The finding reveals that the division of labor in rural development reflects both continuity and change in gender roles. Women remain primarily engaged in activities traditionally associated with domestic and caregiving responsibilities, such as food preparation, small-scale processing, and community welfare, reinforcing established gender norms. However, evidence also points to women's growing involvement in new fields, particularly in small-scale entrepreneurship, fisheries processing, and participation in governance forums. These emerging roles suggest that while gender stereotypes continue to shape the distribution of tasks, there is a gradual trend toward expanding women's participation in areas previously dominated by men, signaling potential shifts in the gendered landscape of rural development.

In Ampekale, gendered task allocation is shaped by cultural expectations regarding physical ability. Men are typically assigned heavy labor based on assumptions of strength and craftsmanship, whereas women handle lighter tasks, such as carrying water or preparing food. These practices reinforce long-standing divisions, influencing both inclusivity and efficiency of the system. Encouraging more flexible and equitable participation can enhance productivity and strengthen the social impact of development initiatives.

Table 3 illustrates how gender perceptions shape the assignment of tasks in rural development and infrastructure. Men are typically assigned roles that require physical strength and technical skills, such as digging and constructing irrigation channels. As one male participant explained:

**Table 3:** The reasons for selecting appropriate activities in rural development organizations and infrastructure from a gender perspective.

**Rural infrastructure development activities**	**Men**	**Women**
**Sand and water transfer by using a bucket to the** **infrastructure site**		
Easy work	High	High
Fasten the works	Poor	Moderate
Appropriate activities for gender participation	High	High
Overall	High	Moderate
**To excavate soil, paint walls, scoop cement, carrying rocks,** **climb ladders**		
Strong physic required	High	Moderate
Appropriate activities for men	High	Poor
Craftmanship skills required	Moderate	Poor
Overall	High	Poor
**Providing meal during construction**		
Appropriate activities	High	High
Domestic activities	Moderate	Moderate
Overall	Moderate	Moderate

“*We do the hard work like digging or carrying rocks because people believe men are stronger*.” (Male participant, Binanga Sangkara)

In contrast, women are often assigned tasks such as preparing meals or carrying lighter materials, which community members view as natural extensions of their domestic responsibilities. One woman described:

“*Cooking for the workers or helping to carry water feels like our role, since it is similar to what we do at home*.” (Female participant, Lalang Tedong)

These accounts show how entrenched norms influence not only the division of labor but also the perceived competence and value of men's and women's contributions.

### 3.3 Rural economic activities: a gender-based analysis

Gender roles shape business activities in local economic organizations. Men handle early production, such as fishery culture and crab catching, which require strength and outdoor work, and market raw materials. Women lead the processing of crab meat, making crackers, packaging, marketing, and running home-based enterprises. These roles reflect traditional norms that link men to production and public work and women to processing and domestic tasks. However, both make essential, complementary contributions, underscoring the need to recognize and support their roles in inclusive rural economic development.

Table 4 demonstrates a clear gendered division of labor within the local economic value chain, where men are more engaged in the production phase and women play central roles in processing and marketing. Men typically undertake physically demanding tasks such as fishing and crab catching, which are perceived as requiring strength and extended time outdoors. As one male participant explained:

**Table 4:** Business types and management practices according to gender perspectives.

**Business activity types**	**Men**	**Womens**
Fishery culture	Production Marketing	Marketing
Crab processed enterprise	Raw material procurement Marketing	Processing Packaging Marketing
Crab catching	Fishing	Provision of fishing operation needs
Seaweed farm	Production Marketing	Processing Marketing
Cracker processed enterprise	Raw material procurement	Production Marketing

“*Going out to sea or catching crabs is our job, it needs strength and time away from home*.” (Male participant, Binanga Sangkara)

By contrast, women are responsible for value-adding processes such as refining crab meat, producing crackers, and managing packaging and sales. A woman emphasized:

“*We work from home processing and selling products, so we can look after children and earn income at the same time*.” (Female participant, Lalang Tedong)

The finding highlights men's and women's distinct but complementary roles shaped by cultural expectations: men focus on raw material procurement, whereas women manage home-based enterprises, processing, finances, and marketing. Women's ability to balance business and household duties reflects their strong organizational skills, which add significant value to local economies. These interdependent roles show how gender dynamics structure rural businesses in India. Both contributions are vital; however, women's roles remain undervalued. Recognizing women's central role in value-adding and marketing, alongside men's role in production, can foster inclusivity and strengthen community development.

Table 4 further illustrates this division: men dominate production tasks such as fishing and material procurement, whereas women lead processing and marketing, refining crab meat, making crackers, packaging, and sales. This complementarity underscores the need to value both roles, as collaboration between genders sustains local businesses and supports equitable rural economic growth.

Gender roles also influence business activities within local economic organizations. Men are often responsible for the initial stages of production, such as fishery culture and crab catching, because these tasks are seen as requiring physical strength and outdoor labor. As one man noted:

“*Going out to sea or catching crabs is our job, it needs strength and time away from home*.” (Male participant, Binanga Sangkara)

By contrast, women lead processing activities, including refining crab meat, making crackers, and managing packaging and marketing. A woman emphasized:

“*We work from home processing and selling products, so we can look after children and earn income at the same time*.” (Female participant, Lalang Tedong)

These perspectives indicate that while men dominate production, women play central roles in value-adding and home-based enterprises, highlighting complementary but unequal contributions shaped by cultural expectations (Cornwall and Goetz, 2005).

This study highlights the complementary roles of men and women in local economic organizations and shows how gender shapes business activities. Women excel in home-based enterprises—processing crab meat and crackers, managing finances, and handling administration—while balancing household duties, reflecting their strong organizational skills. Men focus on labor-intensive tasks, such as fishing and crab catching, dominating early production stages, while women are central to value-adding and marketing. These roles reflect traditional norms, with men supplying raw materials and women managing the processing and sales. Recognizing these contributions enables organizations to harness both strengths, improving effectiveness and fostering sustainable rural development in the process.

Table 5 shows that men are responsible for physically demanding tasks such as digging, construction, and transporting heavy materials, whereas women contribute mainly to supportive roles, including food preparation and carrying lighter items such as sand and water.

**Table 5:** Appropriate business activities through the lens of gender perspectives.

**Activities/activities elements**	**Men**	**Women**
**Boards of LEO**		
Small/household scale business	Moderate	High
Matched with business management	Moderate	High
Support gender mainstreaming of rural development program	Moderate	High
Overall	Moderate	High
**Refined crab meat and cracker processing**		
Matched with production processing	High	High
Related to domestic tasks	Moderate	High
Home industry	Moderate	High
Overall	Moderate	High
**Business management**		
Need a good handwriting	Poor	Moderate
Home business management	High	Moderate
Thorough the jobs	Moderate	Moderate
Work more orderly	Moderate	High
**Financial management**		
Meticulous tasks	Moderate	Moderate
Financial management	High	High
Overall		
**Marketing crackers within the village**		
Activities done at home	Moderate	Moderate
Dominantly stay at home	Poor	Poor
Overall	Moderate	Moderate

Participants emphasized that the division of labor in rural development activities reflects long-standing cultural norms about gender responsibilities. Men are widely perceived as being naturally suited to physically demanding tasks, such as carrying stones, digging, and construction, because these activities are associated with strength and masculine duty. Within this normative framework, women are generally not expected to perform heavy labor, and their exclusion from such tasks is understood as being appropriate rather than discriminatory.

Women's participation is framed as complementary and supportive, closely aligned with their everyday domestic roles. As one female participant explained,

“*When the men are busy with construction, we cook and sometimes help carry small things like water or sand. That is how we support the project*.” (Female participant, Lalang Tedong)

Another woman highlighted the underlying perception of physical strength and suitability, noting that

“*People believe men are stronger, so they handle heavy work. Women help in ways that match our usual responsibilities, like cooking or preparing needs*.” (Female participant, Binanga Sangkara)

By integrating these qualitative accounts with the descriptive patterns presented in Table 5, the findings illustrate not only how labor is divided between men and women but also how cultural norms and gendered assumptions about capability actively reproduce and legitimize this division. Therefore, participation in rural development is structured less by formal rules than by deeply embedded social expectations that shape the work considered appropriate for each gender.

### 3.4 The outcome of community development activities

#### 3.4.1 The outcomes of gender-informed local development initiatives

The finding shows clear gender differences in the perception of local development organizations (LDOs). Women see LDOs as vital for individual and community wellbeing, highlighting expanded access to resources, healthcare, education, and opportunities for participation in decision-making. For many, involvement in LDOs provides a pathway to greater agency and empowerment that extends beyond economic benefits.

In contrast, men focus on material outcomes, such as higher income, business capital, and better infrastructure. Their engagement often reflects expectations of short-term returns tied to production and public decision-making. While they acknowledge the community value of LDOs, men prioritize financial and productivity benefits.

These contrasting views underscore the need for gender-sensitive strategies in rural development. Integrating women's social and welfare concerns with men's economic priorities can create more inclusive and sustainable initiatives. By addressing distinct needs and contributions, programs become more equitable and responsive to community realities.

Table 6 shows clear gender differences in rural development outcomes. Women report direct benefits from involvement in local development organizations (LDOs), especially in healthcare, education, and decision-making, which enhance their empowerment and influence. Men, however, value community participation in broader terms without noting comparable personal gains. This contrast highlights the need for development initiatives to address gender-specific experiences and outcomes.

**Table 6:** The impact of gender-based perspectives on various aspects of rural development.

**Impacts of organization/ activity elements**	**Men**	**Women**
**Rural development activities**		
Increased gender participation especially in problem identification and decision making	Poor	Poor
Increased community participation in rural development activities	High	Moderate
Rural development programs based on community needs and proposals	Moderate	Moderate
Faster public services by local government	Poor	Poor
Overall	Moderate	Moderate
**Community participation in rural development activities**		
Lack of rural infrastructure	Moderate	High
Strong social bond	Moderate	High
Voices are heard	Poor	Poor
Programs related to fulfilling their basic needs	Moderate	Moderate
Overall	Modearte	Moderate
**Perceived improvement of rural people's lives**		
Health services	Poor	High
Educational services	Poor	High
Increased economic activities	Moderate	Moderate
Overall	Poor	High

#### 3.4.2 Gender perspectives in LEO: outcomes and implications for development

The impact of LEOs on rural quality of life shows clear gender disparities. Women report greater benefits in areas of human capital, particularly in supporting children's education and meeting household needs. LEO participation strengthens their ability to manage domestic responsibilities and contribute socially, enhancing both personal wellbeing and community progress. These finding stresses the need to integrate gender considerations in evaluating and implementing LEO projects to ensure equitable outcomes.

Table 7 highlights gender differences in the impact of local economic organizations (LEOs) on rural communities. Women emphasized gains in human capital, especially better access to children's education and meeting household needs, which enhance family welfare and signal growing autonomy in household and community roles. In contrast, men associated LEOs with material achievements, such as business capital or vehicles, reflecting a focus on financial independence and conventional economic success.

**Table 7:** The impact of local economic organizations (LEO) on the quality of life in rural communities from a gender-based perspective.

**Improvement human live elements**	**Men**	**Women**
Sending children to school	Moderate	High
Fulfilling daily needs	Moderate	High
Having their own business capital	Moderate	Poor
Buying a motorcycle with credit	High	Moderate
Having a bank account	High	High
Renovating house	Poor	Poor
Overall	Moderate	Moderate

However, women's broader participation is constrained by structural barriers. Limited access to capital prevents scaling up enterprises such as crab processing or cracker production, while few training opportunities in technical, financial, and marketing skills restrict diversification and product improvement. Unequal access to land, fishing gear, and processing technologies—resources largely controlled by men—further entrenches disparities, weakening women's competitiveness and reinforcing dependence, despite their vital role in value-adding.

Control over income and economic decisions remains uneven. Men typically dominate larger household and community investments, even when income comes from women's work. However, some women reported autonomy over earnings from small enterprises, particularly for daily expenses and children's education. This shows that while women's authority in strategic decisions is limited, their independent income is gradually expanding their decision-making power in everyday domains.

## 4 Discussions

### 4.1 Gendered composition and participation in local development organizations

The finding indicates that the VRB holds significant potential as a platform for inclusive development, but its effectiveness is constrained by persistent gender imbalances. Prior studies emphasize that participatory institutions like VRB can improve accountability and community responsiveness when they incorporate diverse voices (Antlöv et al., 2016; Made Denik Puriati, 2021). However, as shown in Ampekale, women's representation often remains symbolic rather than substantive, echoing broader critiques of decentralization and gendered governance (Cornwall, 2008; Desai et al., 2022; Kabeer, 1999; Made Denik Puriati, 2021; Rahmatunnisa and Mariana, 2017). Strengthening gender-inclusive policies within such institutions is therefore essential, and international evidence suggests that reforms such as quotas, gender-sensitive facilitation, and participatory budgeting can improve women's ability to influence decision-making processes (Mansuri and Rao, 2013; Deepthi and Kettler, 2024). By situating the study's finding within this broader scholarship, the results affirm that while VRB is structurally positioned as a promising space for community participation, deliberate interventions are required to ensure women's contributions translate into meaningful influence rather than symbolic presence.

Prior studies emphasize that participatory institutions like VRB can improve accountability and community responsiveness when they incorporate diverse voices (Antlöv et al., 2016; Made Denik Puriati, 2021). However, as shown in Ampekale, women's representation often remains symbolic rather than substantive, echoing broader critiques of decentralization and gendered governance (Cornwall, 2008; Made Denik Puriati, 2021; Rahmatunnisa and Mariana, 2017). Strengthening gender-inclusive policies within such institutions is therefore essential, and international evidence suggests that reforms such as quotas, gender-sensitive facilitation, and participatory budgeting can improve women's ability to influence decision-making processes (Kettler, 2022; Mansuri and Rao, 2013). By situating the study's finding within this broader scholarship, the results affirm that while BPD is structurally positioned as a promising space for community participation, deliberate interventions are required to ensure women's contributions translate into meaningful influence rather than symbolic presence.

The male-dominated composition of the Village Representative Board (BPD) in Ampekale, where only two of the nine members are women, reflects a persistent gender imbalance in rural governance across Indonesia. Such underrepresentation reduces the diversity of perspectives in decision-making and weakens inclusive governance, especially in addressing women's needs in community development (Van Klinken and Berenschot, 2014; van Klinken and Berenschot, 2018). These finding echoes Cornwall (2008) argument that decentralization often produces symbolic rather than substantive gender inequity. More recent studies show that entrenched patriarchal norms, informal networks, and restricted access to political education continue to privilege male elites and sideline women from leadership pathways (Antlöv et al., 2016; Boasinke, 2021; Cornwall, 2008).

Participatory mechanisms, such as *Musrenbangdes*, in principle, encourage grassroots inclusion, with a quota of 30% female participation. However, in practice, this target remains aspirational. Women's participation is often limited to welfare or household concerns, whereas men dominate debates on infrastructure and economic issues (Syukri, 2021). This reflects what Chaikin and Kirieieva (2020) describe as “instrumental participation,” where women's voices are acknowledged but restricted to traditionally feminine domains. Despite this, the finding in Ampekale shows that women actively propose initiatives in education and healthcare, highlighting their potential for meaningful contributions if institutional practices become more inclusive (Awuni et al., 2022).

These findings align with broader critiques of decentralization and gendered governance, where women's roles in rural institutions are constrained by structural and cultural barriers to women's participation. Patriarchal norms that privilege male leadership, the double burden of domestic and productive responsibilities, and limited access to political education and leadership training continue to restrict women's opportunities for strategic participation (Esfahani and Bahramitash, 2015; Cornwall, 2008). The Ampekale case demonstrates how such barriers reproduce gendered inequalities in governance, limiting the transformative potential of participatory institutions in the village.

Simultaneously, prior research emphasizes that participatory bodies like the BPD can strengthen accountability and responsiveness when diverse voices are included (Antlöv et al., 2016; Syukri, 2023). International evidence further suggests that reforms such as gender quotas, gender-sensitive facilitation, and participatory budgeting are effective in enhancing women's agency and decision-making power (Rahmatunnisa and Mariana, 2017; van Kempen, 2014). Situating the study's findings within this scholarship, this paper argues that while the BPD is structurally positioned as a promising platform for inclusive rural development, deliberate interventions are required to shift women's participation from symbolic presence toward substantive leadership, thereby advancing gender equity and community empowerment in rural governance.

These findings support the call for gender-responsive governance reforms that move beyond quotas and numerical representations. Strengthening participatory planning requires gender-sensitive facilitation, close monitoring of participation quality, and mechanisms that ensure that women's input influences broader development priorities. Recent studies suggest that reforms such as quotas, participatory budgeting, and leadership mentoring can significantly enhance women's agency in local governance (Cornwall, 2008; Rakotoarison, 2024; Yount et al., 2024). Embedding these reforms can transform village organizations, such as the BPD, into more inclusive and equitable institutions, promoting resilience and fairer development outcomes.

### 4.2 Gender roles in rural development activities

The division of labor in rural infrastructure projects in Ampekale highlights how entrenched gender norms shape community participation and the allocation of resources. Men are primarily involved in physical and technical tasks, such as digging, lifting, and construction, activities strongly associated with cultural notions of masculinity and strength (Elmhirst, 2012; Resurreccion and Elmhirst, 2012; Julia and White, 2012). In contrast, women are generally assigned supportive roles, such as preparing meals or transporting lighter materials, including water and sand. These patterns reinforce domestic associations and restrict women's influence on core developmental decisions. Similar dynamics have been observed in Southeast Asia and elsewhere, where infrastructure-related work is gendered and women's contributions are often undervalued or perceived as supplementary rather than strategic (Awuni et al., 2022; Lomazzi, 2023; Rathgeber, 2008).

Although these roles may appear complementary, they expose structural inequalities that marginalize women from visible and valued participation. While women's contributions are essential for sustaining development activities, they often remain invisible in official records and planning processes. This invisibility reduces women's opportunities to access skills training or leadership platforms, thereby perpetuating gender hierarchies within community-based programs (Cornwall, 2008; Han et al., 2024). Recent studies have noted that systematic exclusion from technical and leadership roles limits women's ability to influence priorities that directly affect household and community well-being (Boasinke, 2021; Rakotoarison, 2024; Yount et al., 2024). Addressing these entrenched inequalities requires not only recognizing women's roles but also restructuring practices and institutional mechanisms to ensure equitable access to resources, capacity building, and leadership opportunities.

This study reinforces the notion that gender equity in rural development cannot be achieved without dismantling these structural barriers. Women's participation in rural communities is often confined to supportive or domestic activities, which restrict their visibility in governance and decision-making (Kabeer, 1999; Resurreccion and Elmhirst, 2012). Our finding echos this trend, showing that women's involvement in economic activities such as crab processing and small-scale trading is largely informal and lacks institutional support for advancing leadership. The absence of structured programs, whether from local organizations or external donors, underscores a critical gap in promoting women's strategic participation. Without targeted interventions, women's roles risk remaining symbolic rather than transformative, as they continue to be excluded from leadership in both governance and market systems (Agarwal, 2018; Awuni et al., 2022; Cornwall and Edwards, 2016). Evidence suggests that initiatives such as participatory budgeting, leadership mentoring, and inclusive training programs can create pathways for women to transition from contributors to planners and decision-makers (Cornwall, 2008; Yount et al., 2024). Therefore, promoting women's substantive inclusion is both a matter of equity and a necessary condition for fostering empowerment and achieving inclusive rural development outcomes (Belaid et al., 2021; Shortall, 2002).

### 4.3 Gendered division of labor in local economic organizations (LEOs)

The gender-based segmentation observed in local economic organizations (LEOs) in Ampekale village reveals a clear division of labor shaped by long-standing socio-cultural norms. Men predominantly engage in physically demanding, sea-based production tasks, such as fishing and crab catching. In contrast, women play a central role in processing, packaging, and marketing crab-based products, activities typically conducted near or within the home. This pattern echoes the earlier findings by Elmhirst (2012); Resurreccion and Elmhirst (2012) and Rigg (1998) and aligns with evidence across Southeast Asia that women occupy indispensable, though often less visible, positions in aquaculture value chains (Amandaria et al., 2025; Bosma et al., 2019; Kruijssen et al., 2016; Perret and Yuerlita, 2014).

While mirroring entrenched cultural expectations, this segmentation simultaneously opens avenues for women's empowerment by embedding income-generating activities into the household sphere. Home-based enterprises, such as crab meat processing and cracker production, allow women to contribute to household income while balancing domestic responsibilities, thereby strengthening their agency and social status (Baden, 2013; Kabeer, 1999; Njuki et al., 2022; Quisumbing et al., 2021). These findings support Resurreccion (2006) argument that such engagement enhances women's bargaining power and influence in household decision-making, even when their contributions are not publicly recognized as entrepreneurial.

Consequently, gendered labor division should not be viewed solely as a constraint but also as a potential entry point for inclusive rural development. When adequately supported with training, technology, and credit, women's roles in processing and marketing can become strategic nodes for economic transformation, rather than merely reinforcing tradition (Acosta et al., 2021; Oduol et al., 2017). LEOs can thus move beyond symbolic inclusion by creating institutional mechanisms that foster shared responsibilities, ensure women's access to productive resources, and increase the visibility of their contributions. Such approaches resonate with global discussions on gender-transformative interventions that tackle structural barriers while enhancing women's capabilities (Cornwall, 2008; Doss et al., 2020; Yount et al., 2024).

This research further highlights variations in women's control over income within LEOs. Although women contribute through processing, snack production, and trading, their authority over income allocation is often mediated by male household heads, particularly for larger investments, reflecting the persistent gendered hierarchy in financial governance (Agarwal, 2018; Tavenner and Crane, 2019). However, many women report greater autonomy in managing earnings directed toward daily consumption and children's education. This uneven pattern demonstrates that while women's financial control remains constrained in strategic decisions, it is expanding in domains tied to independent income streams, similar to trends documented in other rural contexts (Belaid et al., 2021; Rakotoarison, 2024). These shifts underline the importance of interventions that enhance women's income opportunities and promote gender-equitable decision-making frameworks at the household and community levels.

### 4.4 Business roles and management: gendered capabilities and perceptions

This study reveals a clear gendered division in business roles and management practices within local economic organizations (LEOs), where women demonstrate strong capabilities in managing home-based enterprises, overseeing financial transactions, and managing administrative responsibilities. These findings echo earlier work on women's aptitude for multitasking and resource management in contexts where household and entrepreneurial roles intersect (Elmhirst, 2012; Resurreccion and Elmhirst, 2012; Mayoux, 2001). In Ampekale, women's engagement in crab meat processing and cracker production, coupled with their control over budgeting and logistics, illustrates both economic efficiency and everyday empowerment, even within the constraints of gender norms. Conversely, men remain more engaged in physically intensive production activities, such as fishing and crab harvesting. This division reflects enduring gender stereotypes that associate men with strength-based labor and women with domestic or supportive functions, thereby reaffirming entrenched perceptions of “suitable” work for each gender (Chant and Sweetman, 2012; Doss et al., 2018).

Despite the persistence of these norms, women's participation in LEOs has generated significant economic and social benefits for the community. Women's enhanced control over enterprise revenues, greater role in household decision-making, and growing visibility in community-level economic affairs signal a gradual transformation in gender dynamics. These outcomes resonate with recent scholarship documenting how women's engagement in hybrid domestic-commercial enterprises can strengthen their bargaining power and reshape intra-household relations (Cornwall, 2008; Garcia and Wanner, 2017; Quisumbing et al., 2021). Moreover, women's entrepreneurial roles contribute not only to household welfare but also to community resilience by diversifying income sources and embedding women's labor into local value chains (Belaid et al., 2021; Rakotoarison, 2024).

Positioned within feminist economic critiques, these findings underscore the importance of recognizing women's productive and reproductive contributions as central to rural economies. By highlighting women's business acumen and their ability to manage enterprises that blur the boundaries between the domestic and commercial spheres, this study supports arguments for gender-transformative approaches to rural development (Yount et al., 2024). Local economic organizations can serve as platforms for institutional change by expanding women's access to credit, training, and leadership opportunities. Strengthening these mechanisms can help LEOs evolve into inclusive economic spaces that challenge entrenched stereotypes and promote equitable development.

### 4.5 Outcomes and impact of gender-informed development initiatives

The outcomes of gender-informed development initiatives reveal a stark divergence in how men and women perceive the benefits and purposes of local development organizations (LDOs). Women in Ampekale Village often view LDOs as critical avenues for empowerment, granting them improved access to healthcare, education, and opportunities for decision-making within the community. This resonates with Kabeer (1999) classic conceptualization of empowerment as the expansion of choice and agency for marginalized groups, while also aligning with more recent scholarship showing that women's participation in community-based organizations strengthens both material wellbeing and social recognition (Cornwall and Edwards, 2016). Women's narratives emphasize not only tangible improvements in household welfare but also symbolic gains in autonomy and visibility, echoing findings that gender-aware initiatives can shift entrenched social norms and gradually redefine women's roles in both public and private domains (Doss et al., 2018; Yount et al., 2024).

By contrast, men's interpretations of LDO benefits are more closely tied to material outcomes such as access to productive assets, income stability, and infrastructure development. This economic-centered view reflects conventional expectations of masculinity that emphasize provision, productivity, and financial control (Chant and Gutmann, 2002). Similar patterns have been observed in rural development programs globally, where men often prioritize economic returns while women highlight improvements in household welfare and social services (Aberman et al., 2018; Belaid et al., 2021). While men and women acknowledge the value of LDOs, their engagement levels and perceived benefits remain shaped by divergent priorities. Women's deeper appreciation for capacity-building and social services contrasts with men's emphasis on economic gains, suggesting that interventions are received and utilized differently across gender lines.

These findings reinforce the importance of gender-sensitive evaluation frameworks that account for these distinct dimensions of development impact. This supports Molyneux (2006) argument that women's empowerment should be assessed not only through economic indicators but also through shifts in agency, wellbeing, and relational power. Recent studies similarly stress that evaluations should capture both tangible and intangible dimensions of empowerment, including changes in self-confidence, decision-making power, and social recognition (Cornwall and Edwards, 2016; Rakotoarison, 2024). This study thus positions gender-responsive LDOs not merely as vehicles for service delivery but as instruments of social transformation. To maximize inclusivity and sustainability, development initiatives should integrate differentiated strategies that reflect gendered experiences, thereby enhancing both their effectiveness and long-term social impact (Njuki et al., 2022; Quisumbing et al., 2021; Yount et al., 2024).

The outcomes of gender-informed development initiatives reveal stark divergence in how men and women perceive the benefits and purposes of local development organizations (LDOs). Women in Ampekale Village view LDOs as critical avenues for empowerment, granting them improved access to healthcare, education, and opportunities for decision-making within the community. This aligns Kabeer (1999) conceptualization of empowerment as the expansion of choice and agency, particularly among individuals who have historically been marginalized. Women's narratives emphasize not only tangible improvements in their household wellbeing, but also the symbolic gains in autonomy and recognition, echoing finding from Cornwall and Edwards (2016), who argue that gender-aware initiatives can shift social norms and redefine women's roles in public and private spheres.

Conversely, men's interpretations of LDO benefits are more closely aligned with material outcomes such as increased access to business assets, income stability, and infrastructure development. This economic-centered view reflects conventional expectations of masculinity tied to provision and financial control (Chant and Gutmann, 2002). While men and women acknowledge the contributions of LDOs, their engagement levels and perceived benefits are shaped by differing priorities. Women's deeper appreciation for social services and capacity-building programs contrasts with men's emphasis on productive investments, suggesting that development interventions may be received and utilized differently across gender lines.

This study reinforces the need for gender-sensitive evaluation frameworks that capture these distinct dimensions of the development impact. This supports Molyneux (2006) argument that women's empowerment should be assessed not only through economic metrics but also by evaluating shifts in agency, wellbeing, and relational power. The findings posit that gender-responsive LDOs are not only vehicles for service delivery but also instruments for social transformation. Therefore, development initiatives should integrate differentiated strategies that acknowledge and respond to gendered experiences to enhance inclusivity, effectiveness, and long-term sustainability.

### 4.6 Implications for inclusive and sustainable rural development

Ampekale's findings underscore the critical role of gender-inclusive planning in advancing sustainable rural development in India. Recognizing and integrating the distinct contributions of men and women, whether in infrastructure projects, local economic organizations (LEOs), or governance, leads to more equitable and responsive development outcomes. As Sharma (2013) emphasizes, inclusive planning that values women's voices not only promote social justice but also strengthens the efficiency and legitimacy of collective decision making. In this study, women's active participation in planning forums and their engagement in home-based enterprises demonstrate their capacity to contribute meaningfully when institutional spaces are provided for them. This affirms Cornwall (2008) assertion that genuine participation requires more than numerical representation, demanding structural reforms that redistribute power and influence. More recent work reinforces this point, showing that gender-inclusive governance mechanisms generate more innovative solutions and equitable resource allocation in rural settings (Belaid et al., 2021).

Gender-sensitive approaches to development have also been shown to enhance program sustainability and foster stronger community buy-ins. Evidence suggests that when women prioritize domains such as healthcare, education, and food security, interventions generate longer-lasting and more socially embedded impacts (Desai, 2010). In Ampekale, the alignment of local development organizations (LDOs) and LEOs with gendered needs has empowered women socially and economically, while strengthening household resilience and community cohesion. This dual impact underscores that gender mainstreaming is not peripheral but a strategic imperative for effective rural development in India. Recent research has further demonstrated that women's leadership in rural cooperatives and development projects not only improves inclusivity but also builds resilience to crises such as climate shocks and economic disruptions (Quisumbing et al., 2021; Yount et al., 2024).

This study supports a policy shift toward embedding gender-responsive frameworks across all levels of rural governance, from village planning forums to cooperative management structures. Such frameworks should incorporate gender-disaggregated data collection, participatory budgeting, leadership training for women, and the continuous monitoring of inclusion metrics. Echoing earlier calls by FAO (2011) and IFAD (2016), but strengthened by more recent scholarship, institutionalizing gender mainstreaming ensures that rural development is both inclusive and adaptive in the face of emerging challenges (Cornwall, 2008; Rakotoarison, 2024). By embedding these practices, rural communities such as Ampekale can advance toward a holistic and resilient development model that fully leverages the contributions of both women and men.

The finding of study underscores the critical role of gender-inclusive planning in advancing sustainable rural development. When the distinct contributions of men and women are recognized and integrated, whether in infrastructure projects, local economic organizations (LEOs), or governance, the result is a process that leads to more equitable and responsive development outcomes. As highlighted by Sharma (2013), inclusive planning that values women's voices not only ensure social justice but also enhances the efficiency and legitimacy of collective decision-making. In this study, women's active participation in planning forums and their engagement in home-based enterprises demonstrate their capacity to meaningfully contribute when institutional spaces are provided. This affirms Cornwall (2008) assertion that meaningful participation requires more than just representation; it also demands structural reforms to redistribute power and influence.

Gender-sensitive approaches to development practices have also been shown to improve program sustainability and community buy-in. For example, when women prioritize healthcare, education, and food security, developmental interventions tend to generate broader and longer-lasting impacts (Desai, 2010). In Ampekale, the alignment of local development organizations LDOs and LEOs with gendered needs has empowered women socially and economically, as well as strengthened household resilience and community cohesion. This dual impact suggests that gender mainstreaming is not a peripheral concern, but a strategic imperative for effective rural development.

This study supports a policy shift toward embedding gender-responsive frameworks across all levels of rural governance, from village planning forums to cooperative management structures. Such frameworks should include gender-disaggregated data collection, participatory budgeting, capacity-building for women leaders, and the ongoing monitoring of inclusion metrics. Echoing the recommendations of the FAO (2011) and IFAD (2016), mainstream gender in local governance ensures that rural development is not only inclusive and just but also adaptive and sustainable in the face of future challenges. By institutionalizing these practices, rural communities such as Ampekale can move toward a more holistic and resilient development model that leverages the full spectrum of local human potential.

## 5 Conclusion and recommendation

This study finds that gender roles in local development and economic organizations are distinct and complementary, yet unequal in influence. Men primarily lead infrastructure projects, production activities, and formal leadership roles within LDOs. Women contribute substantially to welfare support, logistics, processing, and small-scale enterprises in LEOs. However, these contributions are often undervalued or limited to symbolic participation due to entrenched cultural norms and restricted access to decision-making. Simultaneously, women's growing involvement in LEOs, especially in home-based enterprises such as crab processing and marketing, is beginning to shift household bargaining dynamics and increase their community visibility, demonstrating potential pathways toward empowerment. Strengthening women's participation in both types of organizations requires deliberate interventions. These include gender quotas, participatory budgeting, targeted training, and leadership-mentoring programs. Encouraging inclusive forums and formally recognizing women's economic and social contributions can improve equity and strengthen rural resilience. Ultimately, gender-sensitive governance and economic initiatives are critical for ensuring that local organizations foster inclusive and sustainable rural development.

## Data Availability

The datasets presented in this article are not readily available because the dataset contains qualitative information gathered through interviews and focus group discussions. While it is not currently published in a public repository, it may be made available upon reasonable request for academic or research purposes. Requests to access the datasets should be directed to rdarma@unhas.ac.id and ririamandaria@unm.ac.id.
